# Stiffness and ATP recovery of stored red blood cells in serum

**DOI:** 10.1038/s41378-019-0097-7

**Published:** 2019-11-04

**Authors:** Zhensong Xu, Wenkun Dou, Chen Wang, Yu Sun

**Affiliations:** 1 Department of Mechanical and Industrial Engineering, University of Toronto, Toronto, ON Canada; 2 Department of Pathology and Laboratory Medicine, Mount Sinai Hospital, Toronto, ON Canada; 3 Department of Laboratory Medicine and Pathobiology, University of Toronto, Toronto, ON Canada; 4 Institute of Biomaterials and Biomedical Engineering, University of Toronto, Toronto, ON Canada; 5 Department of Electrical and Computer Engineering, University of Toronto, Toronto, ON Canada

**Keywords:** Engineering, Environmental, health and safety issues

## Abstract

In transfusion medicine, there has been a decades-long debate about whether the age of stored red blood cells (RBCs) is a factor in transfusion efficacy. Existing clinical studies investigating whether older RBCs cause worse clinical outcomes have provided conflicting information: some have shown that older blood is less effective, while others have shown no such difference. The controversial results could have been biased by the vastly different conditions of the patients involved in the clinical studies; however, another source of inconsistency is a lack of understanding of how well and quickly stored RBCs can recover their key parameters, such as stiffness and ATP concentration, after transfusion. In this work, we quantitatively studied the stiffness and ATP recovery of stored RBCs in 37 °C human serum. The results showed that in 37 °C human serum, stored RBCs are able to recover their stiffness and ATP concentration to varying extents depending on how long they have been stored. Fresher RBCs (1–3 weeks old) were found to have a significantly higher capacity for stiffness and ATP recovery in human serum than older RBCs (4–6 weeks old). For instance, for 1-week-old RBCs, although the shear modulus before recovery was 1.6 times that of fresh RBCs, 97% of the cells recovered in human serum to have 1.1 times the shear modulus of fresh RBCs, and the ATP concentration of 1-week-old RBCs after recovery showed no difference from that of fresh RBCs. However, for 6-week-old RBCs, only ~70% of the RBCs showed stiffness recovery in human serum; their shear modulus after recovery was still 2.1 times that of fresh RBCs; and their ATP concentration after recovery was 25% lower than that of fresh RBCs. Our experiments also revealed that the processes of stiffness recovery and ATP recovery took place on the scale of tens of minutes. We hope that this study will trigger the next steps of comprehensively characterizing the recovery behaviors of stored RBCs (e.g., recovery of normal 2,3-DPG [2,3-Diphosphoglycerate]and SNO [S-nitrosation] levels) and quantifying the in vivo recovery of stored RBCs in transfusion medicine.

## On the lookout for bad blood

Microfluidic analysis reveals changes in the physical and biochemical properties of stored red blood cells (RBCs) that affect their usefulness for transfusion. The quality of donor RBCs declines in the weeks after collection, affecting their survival and function in transfusion recipients. This degeneration is linked to physical ‘stiffening’ of the cells, and researchers led by Yu Sun of the University of Toronto used a microfluidic device to measure the rigidity of donor RBCs. Sun’s team was particularly interested in whether exposure to physiological conditions restores proper function to refrigerated donor RBCs. They learned that three-week-old RBCs recover normal physical and metabolic function in human serum at body temperature, but six-week-old cells fare much worse. Since six weeks is the official ‘shelf life’ for donor RBCs, these findings suggest reassessment of storage procedures may be appropriate.

## Introduction

According to the World Health Organization, 92 million units of blood are collected annually in 164 countries^1^. Red blood cells (RBCs) are the main type of blood product, with more than 21 million units transfused every year in the United States^2^. Regulations stipulated by the US Food and Drug Administration (FDA) and many other countries specify 42 days as the shelf life for stored RBCs^3^. This “gold standard” was established based on the criterion of posttransfusion RBC survival of 75% or higher after 24 h^4^, where posttransfusion RBC survival was measured by monitoring transfused RBCs labeled with ^51^Cr^5^. However, 25% RBC clearance is orders of magnitude higher than the normal daily RBC clearance ratio (~0.8%) in the human body^6^.

In transfusion medicine, there has been a decades-long debate about whether older RBCs (i.e., RBCs that are stored longer) cause worse transfusion outcomes than fresher RBCs cause^7^. More than 40 clinical studies reported that the use of older RBCs was associated with a significantly increased risk of adverse complications after transfusion^8^. These studies examined the effect of RBC storage on several clinical outcomes, including mortality, rates of infection, and length of a patient’s stay in the hospital ICU^9^. The time point to distinguish fresher and older RBCs was usually 14 days or 21 days^8^. In these studies, patients transfused with older RBCs had higher rates of mortality and infection or longer ICU stays than patients transfused with fresher RBCs.

Results from more recent clinical studies, on the other hand, reported no significant difference in clinical consequences between fresher and older RBCs^10,11^. In two larger-scale clinical trials, transfusion of fresher RBCs compared with older RBCs did not significantly reduce complications of prematurity in very-low-birth-weight infants (*n* = 377) or reduce the rates of organ failure or adverse events among 1098 patients undergoing cardiac surgical procedures^12,13^.

In the human body, RBCs must be transported to every tissue to deliver oxygen; otherwise, tissue hypoxia occurs, potentially resulting in pulmonary hypertension, stroke, and cardiovascular dysfunction^14^. RBCs must also remain in the correct stiffness range to allow them to pass through capillaries in the microcirculation^15^. Higher RBC stiffness result in greater clearance by the spleen and is known to contribute to respiratory distress and systemic sepsis^16^. Clinical research has also identified other disease conditions such as splanchnic ischemia that have developed in patients transfused with older RBCs, which are known to be stiffer than fresher ones^17^. Furthermore, among critically ill patients with sepsis who had older RBCs transfused, sepsis became aggravated^18^. Since septic patients have constricted vessels, insufficiently deformable RBCs can be trapped in the microcirculation, leading to tissue hypoxia and exacerbating patients’ health conditions^19^. ATP concentration in RBCs is also crucial for maintaining RBC physiology. ATP is an organic chemical that provides energy to many processes in RBCs^20^, for instance, actively holding phosphatidylserine to the inner layer of the RBC membrane. A reduced ATP concentration caused by storage^21^ leads to the exposure of phosphatidylserine on the outer layer of RBC membrane^22^, acting as a signal for macrophages to engulf the cells. ATP also plays an important role in regulating the interactions between the spectrin network and the RBC membrane^23,24^. Thus, the depletion of ATP can contribute to RBC stiffness changes during RBC storage.

The storage of RBCs is known to cause cell degradation (e.g., RBC stiffness and ATP depletion), referred to as “storage lesion”^25,26^. For instance, mechanical stiffness changes in stored RBCs have been widely studied^27,28^, and the results consistently revealed that the stiffness of RBCs increases over the storage process. However, the controversy regarding clinical outcomes of transfusing fresher versus older RBCs suggests that the degradation of stored RBCs is reversible and that parameters such as RBC stiffness and ATP levels can recover after transfusion. Therefore, the questions that motivated the present study are whether the stiffness of stored RBCs is reversed under in vivo-like conditions and how storage duration causes differences in recovery from stiffness. Furthermore, since the ATP concentration in RBCs plays an important role in regulating the mechanical stiffness of RBCs^21,26,29^, how well and quickly the ATP level can recover after RBC transfusion also remains to be studied. This paper reports microfluidic measurements of the evolution of stiffness and ATP recovery of stored RBCs under in vivo-like conditions (i.e., in 37 °C human serum) and provides quantitative evidence to answer these questions. The recovery of mechanical flexibility by stored RBCs in human serum was measured with a microfluidic device. The microfluidic approach permits the monitoring of each individual RBC’s stiffness change in the recovery process.

## Materials and methods

The study was performed in accordance with the institutional guidelines for using human tissue samples. Blood samples were collected for routine tests and used for study only after they had undergone all scheduled clinical tests and would otherwise be discarded. The study protocol was approved by the Mount Sinai Hospital Research Ethics Board; informed consent was not required because the samples were selected retrospectively, no patient identifies were disclosed to the study, and the study had no effect on the clinical tests or patient management. Stored blood samples from seven different donors were collected in accordance with a research protocol approved by the Mount Sinai Hospital Review Board. Briefly, blood samples were collected from healthy donors in CP2D anticoagulant. After separation of the plasma and buffy coat, the RBCs were suspended in saline-adenine-glucose mannitol (SAGM) and then stored in a blood bank refrigerator at 4 °C until the experiment was conducted.

**Fig. 1:**
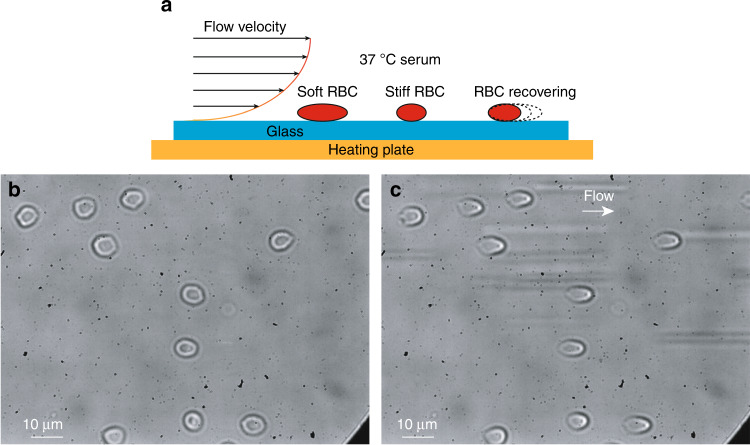
RBCs deformed in the microfluidic device. **a** RBCs strongly adhered to glass substrate after settling for 15 min. RBCs in the microchannel were perfused with 37 °C human serum for 120 min. **b** RBCs in the device without flow. **c** RBCs were deformed by shear flow (flow rate of 10 µL/min). Images of RBC deformation were recorded, and the shear modulus of each RBC was quantified

The microfluidic device was fabricated via soft lithography and PDMS (Polydimethylsiloxane) molding according to our previously reported method^30^. Briefly, the device had a microchannel of 60 µm in height and 1000 µm in width. With the flow rate of 10 µL/min used in our experiments, the generated shear stress of 0.5 Pa is comparable to in vivo conditions^31^, is sufficiently high to deform RBCs (Fig. 1) and is far below the yield stress of RBCs (~100 Pa)^32^. RBCs stored for different periods of time were first diluted 200-fold in PBS and then introduced into the microfluidic channel of the device. The cells strongly adhered to the glass substrate after settling for 15 min^33^. In order to verify the adhesion strength between RBCs and the glass substrate, after 15 min of RBC settling, the applied pressure was varied from 0 to 9 Pa. Throughout the process, no RBC was detached or revealed any noticeable displacement. The RBCs were heated with a heating plate (37 °C, HWPT-384S) throughout the experiments. Human serum (type AB, male, Sigma-Aldrich, glucose concentration 60–140 mg/dL) at 37 °C was continuously perfused in the microchannel for 120 min, and images of RBC deformation (Fig. 1c) were recorded at 1 Hz. Three to seven independent experimental replications were conducted for each tested condition. The shape of each individual RBC was measured via image processing in Matlab, and the shear modulus of each RBC was quantified, according to our previously reported method^30^. Briefly, according to the Kelvin–Voigt (KV) model, $T = \frac{\mu }{2}\left( {\lambda ^2 - \frac{1}{{\lambda ^2}}} \right)$, where *T* (µN/m) is the average tension force acting on the RBC membrane, which was calculated from the shear stress applied on the RBC and the area of the RBC^34,35^; λ is the extension ratio of the RBC membrane, $\lambda = \frac{l}{{l_0}}$, where *l* is the RBC’s length when deformed under shear stress; and *l*_0_ is the RBC’s original length. Each RBC’s elastic shear modulus (*μ*) in force per unit length (µN/m) was determined; a higher shear modulus indicates greater RBC membrane stiffness. Fresh, never-stored RBCs were also tested in the experiment as a benchmark to evaluate how stored RBCs recover their properties in 37 °C human serum. The measured shear modulus is used to quantify the stiffness of RBCs. A higher shear modulus indicates greater stiffness (lower deformability). Statistical analyses were performed using SigmaPlot 13.0. Data are reported as the mean ± standard deviation and were analyzed by one-way ANOVA with Tukey post hoc tests for all pairwise comparisons. The statistical significance of each comparison was evaluated, with significance defined as *p* < 0.05. The recovery of RBCs in this paper specifically refers to the recovery of normal stiffness or ATP levels.

## Results

### Stiffness recovery of stored RBCs in human serum

**Fig. 2:**
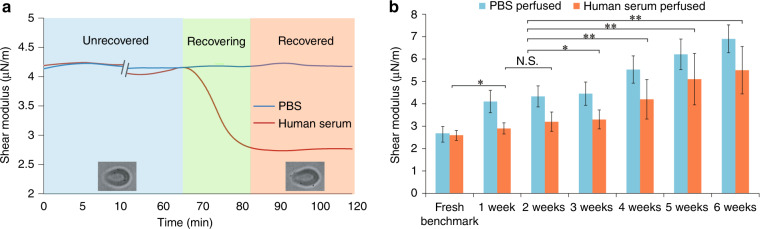
Recovery of stored RBCs’ shear modulus. **a** Shear modulus evolution of 2-week-old RBCs. PBS perfusion (blue line) did not cause RBCs to recover their shear modulus. With perfusion of 37 °C human serum (red line), the RBC shear modulus remained unchanged at 4.3 µN/m for 60 min; however, between 70–80 min, the shear modulus value continuously decreased. By 90 min, a steady state was reached, and the shear modulus became 2.7 µN/m. **b** Steady-state shear modulus values after 120 min of perfusion. With human serum perfusion, RBCs stored for 1 or 2 weeks were able to recover their shear modulus close to the level of fresh RBCs. Older RBCs (4–6-week-old) revealed limited capability of stiffness recovery. *p < 0.05, **p < 0.001. Error bars represent the standard deviation. For each condition, *n* = 1800–3000 RBCs

PBS and human serum were both perfused into the microchannel, and the stiffness changes of individual RBCs were monitored. Figure 2a shows the shear modulus evolution of 2-week-old RBCs (i.e., stored for 2 weeks). During 120 min of perfusion, no stiffness change was observed in the PBS-perfusion group. In the serum-perfusion group, the initial shear modulus of RBCs was the same as in the PBS-perfusion group. It remained unchanged at 4.3 µN/m for ~60 min and then started to decrease. When the shear modulus decreased to approximately 2.7 µN/m, a steady-state was reached, and no further change was observed. These results confirmed that stored RBCs are capable of recovering their shear modulus in human serum, and the recovery process was on the time scale of tens of minutes.

One- to six-week-old RBCs (3000 RBCs from seven different subjects) were tested. Their stiffness after 120-min perfusion periods with PBS and human serum is summarized in Fig. 2b. Since no stiffness recovery was observed from 120-min perfusion in the PBS-perfusion group, the blue bars in Fig. 2b represent the initial RBC stiffness for each storage duration (e.g., initial shear modulus of 2-week-old RBCs: 4.3 ± 0.5 µN/m). For the serum-perfusion group [red bars in Fig. 2b], the RBCs stored for 1 and 2 weeks recovered their shear modulus from 4.1 ± 0.5 µN/m and 4.3 ± 0.5 µN/m to 2.9 ± 0.3 µN/m and 3.2 ± 0.4 µN/m, which are approximately 1.1 and 1.2 times the shear modulus of fresh RBCs (2.6 ± 0.3 µN/m). However, RBCs stored for 4 weeks or longer were able to recover only from 5.5 ± 0.6 µN/m to 4.2 ± 0.9 µN/m (4 weeks), from 6.2 ± 0.7 µN/m to 5.1 ± 1.1 µN/m (5 weeks), and from 6.9 ± 0.6 µN/m to 5.5 ± 1.1 µN/m (6 weeks). These results indicate that RBCs stored for longer than 3 weeks have a limited capacity for stiffness recovery. For instance, 6-week-old RBCs after recovery have twice the shear modulus of fresh RBCs (5.5 ± 1.1 µN/m vs. 2.6 ± 0.3 µN/m), i.e., very poor deformability. Note that for stored RBCs of all ages (1–6 weeks), stiffness recovery was not biased by the shear stress from perfusion (Fig. S1).

### Fresher RBCs become lose stiffness faster

**Fig. 3:**
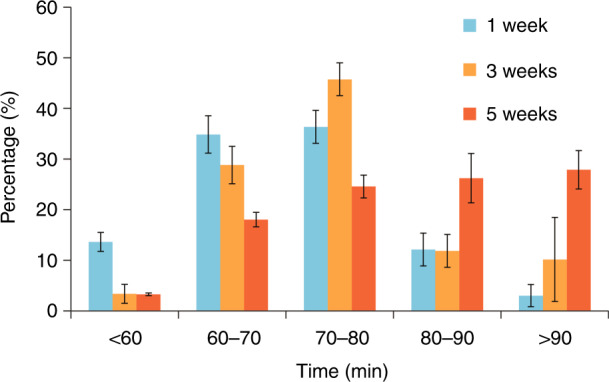
Higher percentages of 1- and 3-week-old RBCs than 5-week-old RBCs reached their steady state shear modulus by 90 min

Fresher and older RBCs also took different amounts of time to reach their steady-state shear modulus. For instance, by 80 min, 85% of 1 week-old RBCs and 80% of 3-week-old RBCs had reached their steady-state shear modulus. In contrast, the percentage of 5-week-old RBCs was only 47% (Fig. 3). Although measurements were made continuously for 120 min, most of the stored RBCs reached their steady-state shear modulus between 60 and 90 min. Between 90 and 120 min, for all the stored RBC samples (1–6 weeks old), almost no RBC revealed a change in its shear modulus. The results (Fig. 3) also show that 5-week-old RBCs took longer than 1- or 3-week-old RBCs to recover their flexibility, and by 90 min of human serum incubation, ~30% of 5-week-old RBCs showed no recovery. This indicates that RBCs stored longer than 5 weeks have a limited capacity to recover from stiffness. We also conducted additional experiments in which we continuously perfused stored RBCs for 8 h (Fig. S2a); we found no further recovery by the RBCs, and the results were consistent with those obtained from the 120-min perfusion experiments. These data show that older RBCs require a longer time to reach a steady-state shear modulus and that a higher percentage of older RBCs cannot recover their stiffness in human serum. We also tested the shear modulus of stored RBCs at both 25 °C (room temperature) and 37 °C. As shown in Fig. S3, no significant difference in the steady-state shear modulus values was found between the 25 °C group and the 37 °C group (see Supplementary Materials for details), indicating that 37 °C alone cannot induce RBC stiffness recovery.

### Shape recovery of stored RBCs in human serum

**Fig. 4:**
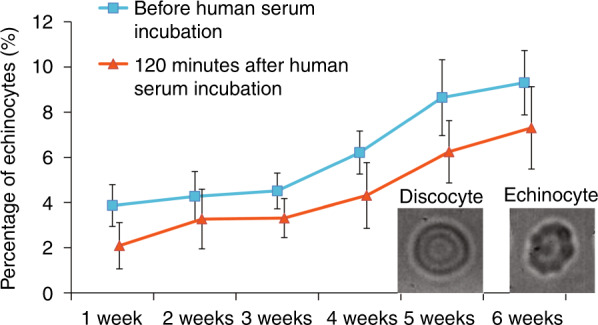
Before incubation in human serum, a higher percentage of echinocytes existed in old RBC samples than in fresher ones. After 120 min of incubation in human serum, the percentage of echinocytes decreased from 3.8 to 2.1% for 1 week-old RBCs, from 4.3% to 3.3% for 2-week-old RBCs, from 4.5 to 3.3% for 3-week-old RBCs, from 6.2 to 4.3% for 4-week-old RBCs, from 8.6 to 6.2% for 5-week-old RBCs, and from 9.3 to 7.3% for 6-week-old RBCs. For each condition, *n* = 2200–2800 RBCs

During storage, RBCs change their shape from biconcave to echinocytes^36^. In order to investigate the shape recovery of stored RBCs (i.e., reversal from echinocytes to biconcave), RBCs were cultured in 37 °C human serum for 120 min. The percentage of echinocytes before and after human serum incubation was measured, as shown in Fig. 4. Before incubation in human serum, the percentage of echinocytes was ~4% for the 1–3 week-old RBCs. The percentage increased to 6% for the 4-week RBCs, and by the end of the storage (i.e., 6 weeks), the percentage had increased to ~9%, agreeing well with previously reported results of storage lesions^36,37^. After 120 min of incubation in human serum, the percentage of echinocytes for 1–3-week-old RBCs decreased to approximately 2%, while a higher percentage of older RBCs remained echinocytes (4%, 6%, and 7% for 4-, 5-, and 6-week-old RBCs, respectively), indicating that RBCs stored for a shorter time period are more capable of recovering their shape from echinocytes back to biconcave.

### ATP concentration recovery of stored RBCs in human serum

**Fig. 5:**
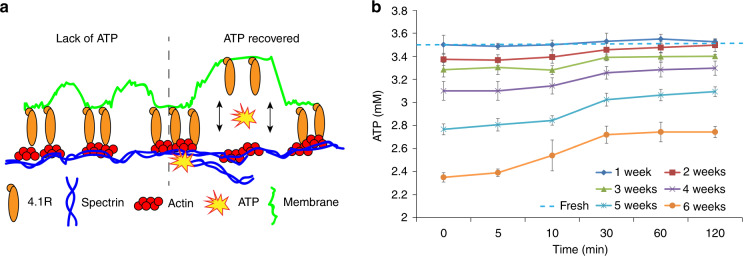
Recovery of stored RBCs’ ATP. **a** Lack of ATP increases the spectrin-membrane affinity, leading to increased RBC stiffness. When ATP is re-synthesized, spectrin-membrane binding becomes dynamic, causing the RBC to become more deformable. **b** ATP concentration in RBCs that were stored for 1–6 weeks. For each data point, *n* = 3 samples with each sample containing approximately 8000 RBCs. Older RBCs showed lower ATP concentrations than fresh RBCs, and their ATP concentrations increased during serum incubation. Steady-state values were reached by ~30 min

The RBC membrane is composed of a phospholipid bilayer tethered to the underlying spectrin network (spectrin α and spectrin β), as shown in Fig. 5a. RBC stiffness is dependent on the interactions between the phospholipid bilayer and the underlying spectrin cytoskeleton^26^. The interactions are regulated by the binding between spectrin and 4.1R, a protein embedded in the phospholipid bilayer^23,24^. Spectrin/4.1R binding is modulated by phosphorylation, which consumes intracellular ATP^29^. In this work, the intracellular ATP concentration of stored RBCs was measured (ATP Bioluminescent Assay kit; Promega, Madison, Wisconsin, United States; see details in Supplementary Materials).

Fresh RBCs and stored RBCs (1–6 weeks) were incubated in human serum at 37 °C. For each time point during serum incubation, ~8000 RBCs were used to quantify average ATP concentration. We also measured the intracellular ATP concentration of fresh RBCs (3.50 ± 0.09 mM) as the control group. As shown in Fig. 5b, 1 week storage did not induce a significant reduction in ATP concentration (3.50 ± 0.08 mM). However, in the RBCs stored for 4 weeks, the ATP concentration was only 3.10 ± 0.08 mM, and in 6-week-old RBCs, it was as low as 2.35 ± 0.04 mM. This ATP degradation is consistent with the values previously reported for RBC storage lesions^21^. Mitochondria are the organelles that use oxygen to synthesize ATP in most types of cells. RBCs, which do not contain mitochondria, synthesize ATP by glycolysis, an oxygen-independent metabolic pathway that converts glucose into lactate. Glycolysis contains ten enzyme-catalyzed reactions, and in two of these reactions (converting 1,3-bisphosphoglycerate to 3-phosphoglycerate and converting phosphoenolpyruvate to pyruvate), ATP is synthesized in the cytoplasm. It is known that RBC storage causes a consistent decrease in glucose over the storage period^21^, resulting in reduced glycolysis activities and thus a reduction in intracellular ATP concentration.

In human serum incubation, an increased ATP concentration in stored RBCs, especially older RBCs (e.g., 4–6 weeks), was already apparent within the first 10 min of serum incubation, and by 30 min, their ATP concertation started to plateau. Longer incubation (8 h) was also conducted, and it was verified that the stored RBCs had already reached a steady-state ATP by 120 min, after which no further change occurred (Fig. S2b). Our data reveal that the ATP concentration of stored RBCs increased in human serum but could not fully recover in the case of older RBCs. For instance, for 2-week-old RBCs, serum incubation fully restored their ATP concentration to that of fresh RBCs, from 3.37 ± 0.05 mM to 3.50 ± 0.05 mM by 120 min. However, the steady-state ATP concentration of 6-week-old RBCs after 120-min serum incubation merely increased from 2.35 ± 0.04 mM to 2.74 ± 0.05 mM (vs. 3.50 ± 0.09 mM for fresh RBCs).

## Discussion

Many clinical studies of RBC transfusion have investigated whether older RBCs cause worse clinical outcomes, but these clinical trials have provided conflicting information^8,38^: some show that older blood is less effective^9^, but others show no such difference^12^. Currently, there are still strong controversies regarding whether the age of stored RBCs is a factor in transfusion efficacy. Intuitively, fresher RBCs may function better than older RBCs because storage is known to induce biomechanical and biochemical degradation of RBCs, with longer storage leading to more severe degradation^27,28^. The controversial clinical trial results could have been biased by the vastly different conditions of the patients involved in the clinical studies; however, the inconsistency is also due to a lack of understanding of how well and quickly stored RBCs can normalize their key parameters, such as stiffness and ATP concentration, after transfusion.

After blood donation and processing, RBCs were placed in preservation medium for storage at 4 °C. The purpose of the low temperature was to keep the rate of glycolysis low and minimize the proliferation of bacteria that might have entered the blood unit^8^. In the preservation medium, citrate functions as an anticoagulant, mannitol is used to prevent RBCs from undergoing swelling/hemolysis, and a strictly standardized amount of glucose is also added into the preservation medium to help maintain metabolism in the RBCs^39^. Over the storage period, the glucose concentration significantly decreases due to glycolysis. This leads to a reduced ATP concentration in stored RBCs^21^ and causes the RBCs’ stiffness to increase, as shown in Figs. 2b and 5b. It should be noted that RBC preservation protocols do not allow a large amount of glucose to be added into the preservation medium, since adding a large amount of glucose can cause compromising events such as glycosylation of the RBC membrane and skeletal proteins^40^.

Storage-induced degradation of RBCs can lead to the clearance of transfused RBCs in vivo. In a healthy human being, only ~0.8% of RBCs are cleared within 24 h^41^; in contrast, a significantly higher percentage of stored RBCs after transfusion are cleared (e.g., 25% for 6-week-old RBCs)^41^. The clearance of RBCs mainly occurs in the spleen^42^, which consists of a meshwork with many tiny pores (e.g., 5 µm)^43^. RBCs with greater stiffness than normal have an increased chance of being trapped within the meshwork and cleared^44^. Stiffness itself has been shown to serve as a signal for macrophages to induce phagocytosis for RBC clearance^45,46^.

When stored RBCs are transfused into the human body, the major microenvironment they encounter is human serum. In this work, we quantitatively studied the stiffness and ATP recovery of stored RBCs in 37 °C human serum. Compared to previous studies, which measured the mechanical degradation of RBCs at discrete time points during storage^30^, the method used in this paper permitted continuous monitoring of the evolution of individual RBCs’ stiffness recovery for at least two hours in an in vivo-like condition. In addition, ATP recovery was also quantified, indicating that ATP could potentially be a key factor for RBC stiffness recovery. The results showed that in 37 °C human serum, stored RBCs are able to recover their stiffness and ATP concentration to varying extents depending on their duration of storage. As summarized in Table 1, one can see that for 1 week-old RBCs, although the shear modulus before recovery was 1.6 times that of fresh RBCs, 97% of the cells had their stiffness recovered in human serum to be 1.1 times that of fresh RBCs, and the ATP concentration of 1 week-old RBCs after recovery showed no difference from that of fresh RBCs. For 3-week-old RBCs, 89% of RBCs recovered their stiffness to 1.3 times that of fresh RBCs, and the recovered ATP concentration was only 5% lower than that of fresh RBCs. However, for 6-week-old RBCs, only approximately 70% of the RBCs showed stiffness recovery in human serum; their shear modulus after recovery was still 2.1 times that of fresh RBCs; and their ATP concentration after recovery was 25% lower than that of fresh RBCs. Overall, the results indicate that fresher RBCs (1–3 weeks) have significantly higher stiffness and ATP recovery capabilities in human serum than older RBCs (4–6 weeks).

**Table 1:** Shear modulus and ATP concentration of stored RBCs before and after recovery in human serum

Age of RBCs	Shear modulus before recovery (µN/m)	Shear modulus after recovery (µN/m)	Percentage of RBCs that recovered (%)	ATP concentration before recovery (mM)	ATP concentration after recovery (mM)
Fresh (benchmark)	2.6 ± 0.3	2.6 ± 0.3	N/A	3.50 ± 0.09	3.50 ± 0.09
One week	4.1 ± 0.5	2.9 ± 0.3	97 ± 2.2	3.50 ± 0.08	3.53 ± 0.02
Two weeks	4.3 ± 0.5	3.2 ± 0.4	95 ± 1.7	3.37 ± 0.05	3.50 ± 0.06
Three weeks	4.5 ± 0.5	3.3 ± 0.4	89 ± 8.3	3.28 ± 0.06	3.39 ± 0.02
Four weeks	5.5 ± 0.6	4.2 ± 0.9	85 ± 5.7	3.10 ± 0.08	3.30 ± 0.06
Five weeks	6.2 ± 0.7	5.1 ± 1.1	72 ± 3.8	2.76 ± 0.05	3.15 ± 0.04
Six weeks	6.9 ± 0.6	5.5 ± 1.1	70 ± 3.2	2.35 ± 0.04	2.79 ± 0.05

**Fig. 6:**
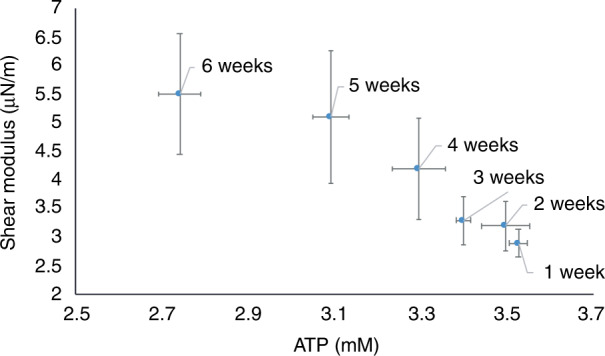
Correlation between shear modulus recovery and ATP concentration recovery. The shear modulus decreased when the ATP concentration increased

Our experiments showed that neither stiffness nor ATP concentration recovered when the stored RBCs were incubated in PBS. The composition of human serum is more diverse than that of PBS^21^. For instance, human serum contains abundant glucose and pyruvate, while these components are absent in PBS^47^. Glucose is converted into lactate by the metabolic pathway of glycolysis. Among the ten reactions involved in glycolysis, 1,3-bisphosphoglycerate is converted to 3-phosphoglycerate, and phosphoenolpyruvate is converted to pyruvate. The subsequent conversion of pyruvate to lactate provides nicotinamide adenine dinucleotide (NAD), which promotes one of the reactions in glycolysis, i.e., the conversion of glyceraldehyde 3-phosphate to 1,3-bisphosphoglyceric acid, and the molecules produced in this reaction are utilized by RBCs to synthesize ATP^48^. Our data quantitatively revealed the recovery of ATP concentration in stored RBCs when they were incubated in human serum. The correlation between the recovered ATP concentrations and the recovered shear modulus values is shown in Fig. 6, showing that the RBCs’ shear modulus decreased when their ATP concentration increased during the recovery process. Since it is known that ATP regulates the tension of the spectrin-membrane connection and thus RBC stiffness^26,29^, it is likely that the stiffness recovery of stored RBCs in human serum is preceded by the recovery of ATP concentration.

Our experiments also revealed that the processes of stiffness recovery and ATP recovery took place on the scale of tens of minutes (Figs. 3 and 5). For instance, for 1 week-old RBCs, the recovered steady-state ATP concentration and stiffness value were reached by 10 and 80 min, respectively. For 6-week-old RBCs, the recovery of ATP concentration and stiffness took 30 min and 90 min, respectively. To put these numbers in context, an RBC completes one cycle of circulation in the human body within a minute^49^. The long recovery time (tens of minutes) could increase the chance for stored RBCs to be cleared by the spleen. This might imply that when feasible in RBC transfusion, pretreatment of stored RBCs in the target patient’s serum might help achieve a higher transfusion efficacy.

RBC degradation (e.g., RBC stiffness and ATP), or storage lesion, occurs during RBC storage^25,26^. The present study focused on investigating whether the degradation could be reversed under in vivo-like conditions (i.e., in 37 °C human serum). Our results indicate that although the degradation of stored RBCs started to occur from the first week of storage, after human serum treatment, stored RBCs were capable of recovering their stiffness and ATP concentration. Our results also show that longer stored RBCs had a limited capacity for recovery; for example, comparing 6-week-old RBCs and 3-week-old RBCs, ~20% of less RBCs recovered in stiffness. The recovered 6-week-old RBCs had a stiffness that was 1.7 times that of recovered 3-week-old RBCs, and the recovered ATP concentration was 20% lower than that of 3-week-old RBCs. This significantly poorer recovery capability may call for a revisit of the policy of storing RBCs for up to 42 days. It should be noted that the RBCs tested in this work were stored at 4 °C. For other RBC storage protocols (e.g., storing RBCs in liquid nitrogen^50^), the recovery behaviors of RBCs might vary from those reported in this paper. We also note that this study used human serum to mimic the in vivo environment, but the environment in the human body after the transfusion is more complex than what serum provides. For instance, can other factors in the human body, such as adrenaline, which is absent in the serum used in our study, speed up the recovery of stored RBCs? We hope that this study will trigger the next steps of comprehensively characterizing the recovery behaviors of stored RBCs (e.g., recovery of normal 2,3-DPG and SNO levels) and quantifying the in vivo recovery of stored RBCs in transfusion medicine.

## Conclusion

This paper reports microfluidic measurements of the evolution of stiffness and ATP recovery of stored RBCs under in vivo-like conditions (i.e., in 37 °C human serum). The results provide quantitative evidence to answer the following questions: whether the stiffness and ATP of stored RBCs can recover in human serum and how storage duration causes differences in the recovery of stiffness and ATP concentration. Despite the degradation induced by storage, stored RBCs were able to recover their stiffness and ATP concentration in human serum. RBCs stored for 1–3 weeks were capable of recovering their stiffness and ATP concentration to values near those of fresh RBCs, while older RBCs (4–6 weeks), even after recovery, had significantly greater stiffness and lower ATP concentrations than fresher RBCs. The results also revealed that the processes of stiffness recovery and ATP recovery occurred on the scale of tens of minutes. These findings provide new insight into how well and quickly stored RBCs recover after transfusion.

## Supplementary information

Stiffness and ATP Recovery of Stored Red Blood Cells in Serum
