# Supplementary material for: Stiffness and ATP recovery of stored red blood cells in serum
Source: Microsyst Nanoeng. 2019 Nov 4;5:51. doi: 10.1038/s41378-019-0097-7 (PMC6826049; doi:10.1038/s41378-019-0097-7)
Supplement: Supplementary file 1 — Stiffness and ATP Recovery of Stored Red Blood Cells in Serum [file 41378_2019_97_MOESM1_ESM.docx]

**Supplementary Materials**

**Stiffness and ATP Recovery of Stored Red Blood Cells in Serum**

*Figure S1. Stiffness of RBCs perfused for 120 minutes in human serum vs. end-point measured stiffness of RBCs incubated in human serum in an incubator without perfusion (n=1300-3000 RBCs for each condition). No difference was observed between the perfusion group and incubating group (*p>0.05), showing that shear stress from perfusion did not induce bias in RBC stiffness recovery.*


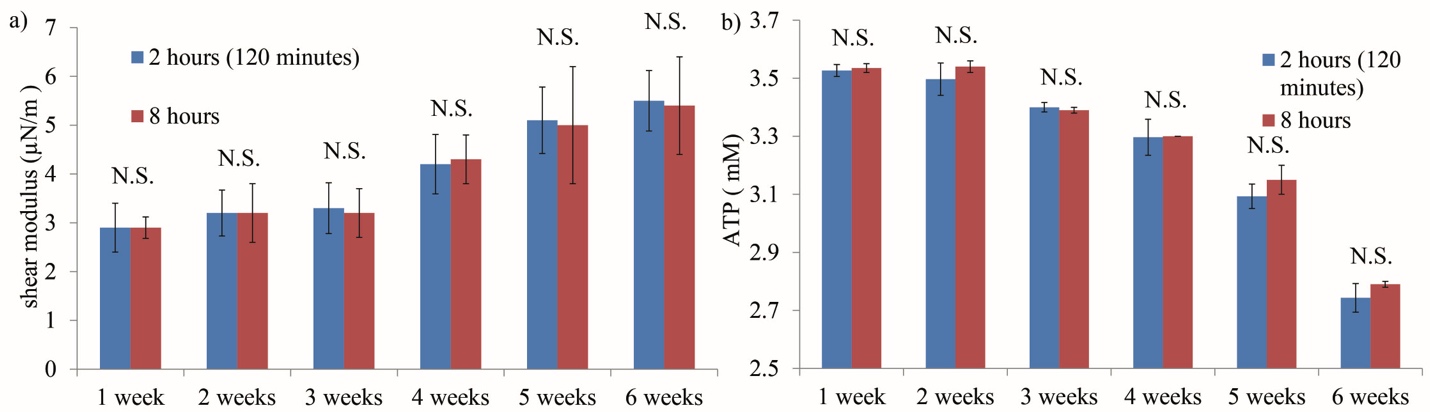


*Figure S2. a) Stiffness of RBCs perfused up to 120 minutes in human serum vs. perfused up to 8 hours (n=790-3000 RBCs for each condition). No difference was observed (*p>0.05), showing that longer perfusion did not induce further recovery of shear modulus. b) RBCs were cultured in human serum for 8 hours. For each sample, the intracellular ATP concentration by 120 minutes and at the end of the 8-hr incubation was compared. For each condition, n=3 samples with each sample containing about 8000 RBCs. For all stored RBCs (one-week to six-week old), no difference was observed, indicating that longer incubation (>120 minutes) did not induce further re-synthesize of ATP.*

*Figure S3. Steady-state shear modulus of stored RBCs that were PBS or serum perfused at 25°C or 37°C (n=1260-3000 RBCs for each condition). The results show that 25°C and 37°C did not cause a statistically significant difference in the stiffness of PBS-perfused or serum-perfused RBCs.*

*
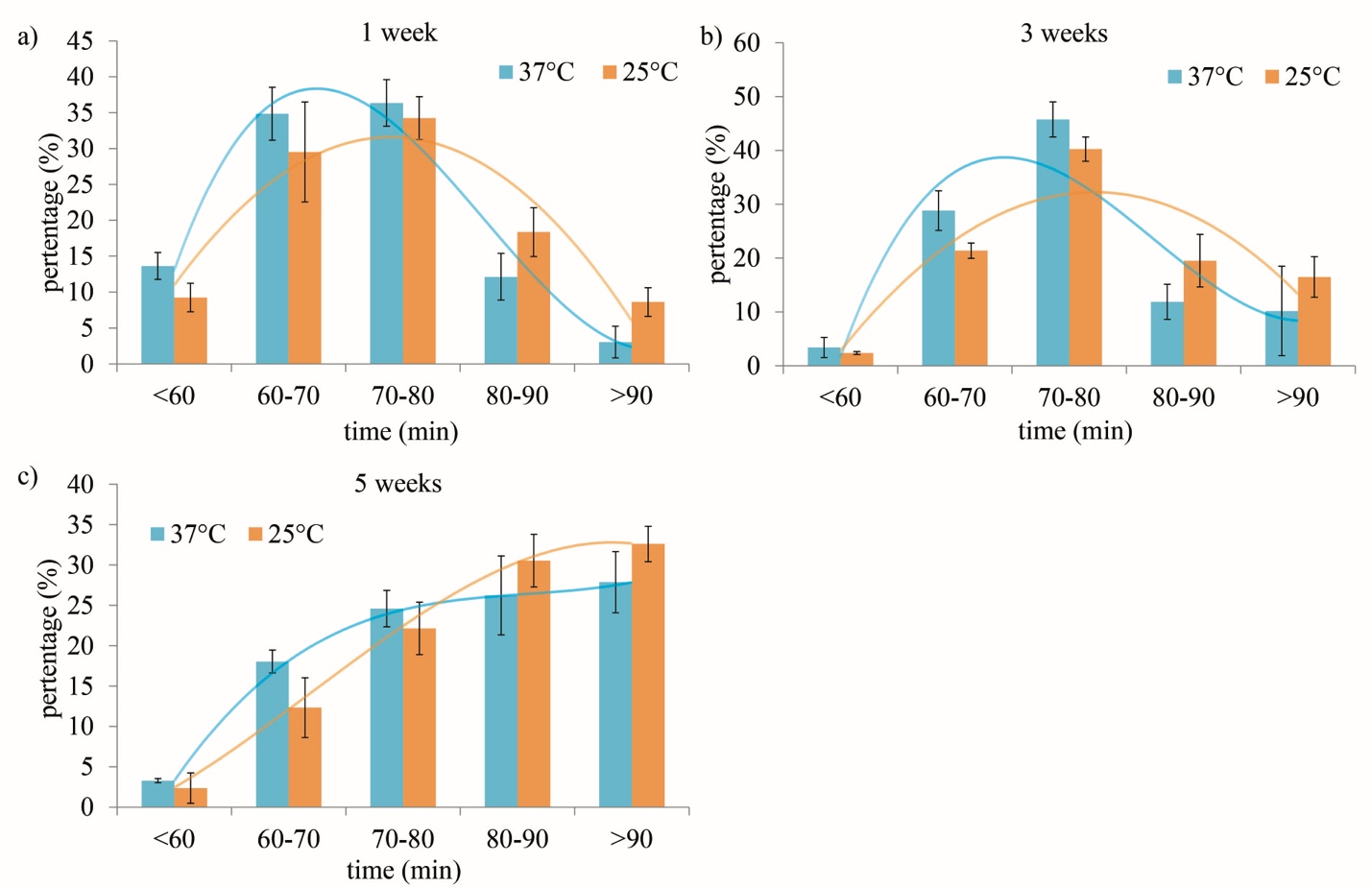
*

*Figure S4. Compared to 25*°*C, 37°C accelerated the stiffness recovery process for stored RBCs in human serum. Second degree polynomial fitting trend lines are also added. Error bars represent the standard deviation.*

**Temperature effect on RBC stiffness recovery comparing room temperature and 37°C**

We tested the shear modulus of PBS-perfused RBCs and serum-perfused RBCs at both 25°C (room temperature) and 37°C. As shown in Figure S3, when the stored RBCs were perfused with PBS, after two hours of perfusion, no significant difference in the steady-state shear modulus values was found between the 25°C group and the 37°C group, indicating that 37°C alone cannot induce RBC stiffness recovery. The lipid tails in the RBC membrane become more unsaturated as temperature increases, resulting in more free space within the lipid bilayer and thus, a more deformable membrane [1]. At 22°C, the stiffness of the RBC membrane has reached the steady state because the lipid tails are fully unsaturated [2]. This phenomenon is consistent with our finding that 25°C and 37°C did not cause a statistically significant difference in the stiffness of PBS-perfused RBCs.

For serum-perfused RBCs, we also found no difference in the steady-state shear modulus between the 25°C group and the 37°C group. However, the RBCs perfused at 37°C showed a faster recovery process than at 25°C. Figure S4 shows the data collected on one-week, three-week and five-week old RBCs. Higher percentages of 37°C RBCs than 25°C RBCs reached their steady-state values by 80 minutes. This can be attributed to higher temperature-caused faster ATP-regulated unbinding of RBC membrane proteins [3]. Our results suggest that, compared to 25°C, 37°C does not produce an additional effect on the stiffness recovery of stored RBCs; however, it accelerates the stiffness recovery process in the human serum environment.

**RBC intracellular ATP concentration measurement**

The Luciferin technique (ATP Bioluminescent Assay kit; Promega, Madison, Wisconsin, United States) was used to measure RBC intracellular ATP concentration. For each sample, about 8,000 RBCs were first washed by PBS at least three times. The RBCs were then lysed by 5% trichloroacetic acid (TCA) to extract ATP in the RBCs. Tris-acetate, as a buffer solution, was added to neutralize the TCA. The rL/L reagent from the assay kit was added into the ATP exacted solution and reacted with ATP to emit light with a wavelength of 560 nm measured by a fluorescence camera. The light intensity was used to quantify the amount of ATP. The assay kit contains a vial of ATP Standard (10-7 M) which was used to construct an ATP standard curve according to the assay kit’s protocol. The ATP standard curve was used as the reference to quantify measured ATP in each experiment. Then the average ATP concentration with a unit of M (mol/L) was calculated from the measured amount of ATP (with a unit of mol) and the total volume of the RBCs (each RBC has a volume of ~90 pL).

**References:**

[1] M. Singh and J. F. Stoltz, “Influence of temperature variation from 5 degrees C to 37 degrees C on aggregation and deformability of erythrocytes.,” *Clin. Hemorheol. Microcirc.*, vol. 26, no. 1, pp. 1–7, 2002.

[2] M. W. Rampling and P. Whittingstall, “The effect of temperature on the viscosity characteristics of erythrocyte suspensions,” *Ciln. Hermorheol.*, vol. 7, no. c, pp. 745–755, 1987.

[3] K. K. Kalsi and J. González-Alonso, “Temperature-dependent release of ATP from human erythrocytes: Mechanism for the control of local tissue perfusion,” *Exp. Physiol.*, vol. 97, no. 3, pp. 419–432, 2012.
